# Self-Assembly of DNA Molecules: Towards DNA Nanorobots for Biomedical Applications

**DOI:** 10.34133/2021/9807520

**Published:** 2021-10-19

**Authors:** Yong Hu

**Affiliations:** ^1^Department of Polymeric Materials, School of Materials Science and Engineering, Tongji University, Shanghai 201804, China; ^2^Institute for Advanced Study, Tongji University, Shanghai 200092, China

## Abstract

DNA nanotechnology takes DNA molecule out of its biological context to build nanostructures that have entered the realm of robots and thus added a dimension to cyborg and bionic systems. Spurred by spring-like properties of DNA molecule, the assembled nanorobots can be tuned to enable restricted, mechanical motion by deliberate design. DNA nanorobots can be programmed with a combination of several unique features, such as tissue penetration, site-targeting, stimuli responsiveness, and cargo-loading, which makes them ideal candidates as biomedical robots for precision medicine. Even though DNA nanorobots are capable of detecting target molecule and determining cell fate via a variety of DNA-based interactions both in vitro and in vivo, major obstacles remain on the path to real-world applications of DNA nanorobots. Control over nanorobot's stability, cargo loading and release, analyte binding, and dynamic switching both independently and simultaneously represents the most eminent challenge that biomedical DNA nanorobots currently face. Meanwhile, scaling up DNA nanorobots with low-cost under CMC and GMP standards represents other pertinent challenges regarding the clinical translation. Nevertheless, DNA nanorobots will undoubtedly be a powerful toolbox to improve human health once those remained challenges are addressed by using a scalable and cost-efficient method.

## 1. Main Text

In nature, DNA molecule is typically used by biological systems to store and transmit genetic information. Over the past decade, DNA nanotechnology takes DNA molecule out of its biological context and use it as fundamental building block to build nanostructures in well-defined yet almost arbitrary sizes and shapes via complementary base-pairing [1], thereby providing a means to tailor nanostructures' biological availability and activity. Tremendous development in assembling functional DNA nanostructures [2] has integrated elementary functions and then elicited various stimuli-responsive mechanisms to resolve daunting tasks in a programmed manner with molecular accuracy. DNA nanostructures have evolved from the initially static state to the increasingly enabled, dynamic state. That is to say, DNA nanostructures have entered the realm of robots and thus added a dimension to cyborg and bionic systems.

Importantly, as a pliable and robust molecule, DNA mechanically behaves like an entropic spring. Hence, it can be elastically stretched and bent by external forces and then reconstitute itself in proper conditions [3]. DNA in the assembled nanostructure retains most of these spring-like properties [4]. This affects the assembly's bending and torsional rigidity along with its inherent architecture. Thus, the mechanical properties of the assemblies can be tuned from wire-like flexibility to beam-like stiffness by deliberate design [4]. Furthermore, stiff beams can be connected by short oligonucleotides to constitute a hinge and its analogue like slider, crank-slider, or Bennet linkage to facilitate an angular motion, linear motion, reversible 3D motion cycle and/or other restricted, mechanical motion [5].

**Figure 1:**
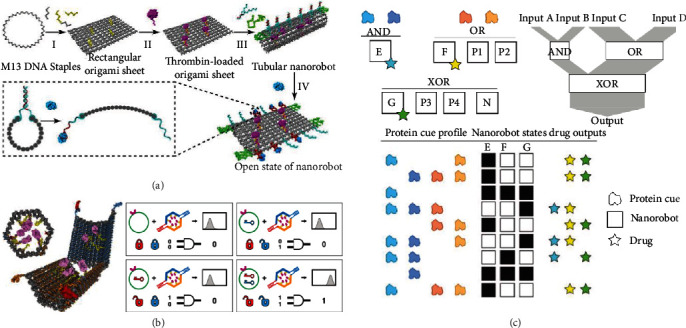
DNA nanorobots for biomedical applications. (a) Design and working principles of the thrombin-loaded tubular DNA nanorobot from which aptamers interact with nucleolin for opening the robot and display of cargo. Reprinted with permission from ref [9]. Copyright 2018 Springer Nature. (b) and (c) The logic gated DNA nanorobots can adopt distinct states to enable different drug outputs depending on the interaction with protein cue profile. (b) Reprinted with permission from ref [10]. Copyright 2012. The American Association for the Advancement of Science. (c) Reprinted with permission from ref [11]. Copyright 2014 Springer Nature.

With delicate design, DNA nanostructures can outperform conventional nanomaterials made of synthetic polymers for diagnostic and sensing applications through their unique features of stimuli responsiveness. For instance, DNA nanorobots constructed with several fluorescent dye-modified oligonucleotides have been used for spatiotemporal mapping of a wide scope of ions (i.e., H^+^, Cl^−^, Na^+^, K^+^, and Ag^+^) based on the conformational transition of i-motif in response to protons or other ions in living cells using Förster resonance energy transfer (FRET) [6–8]. Other than aforementioned targets, ATP, DNA, RNA, and antibody have been detected as well because a variety of DNA-directed specific interactions (i.e., DNA-ATP, DNA-DNA, DNA-RNA, and DNA-protein) [8] can be employed to trigger the closing or opening of DNA nanorobots with sophisticated shapes like plier [8], capsule [9, 10] (Figures 1(a) and 1(b)) and others. The conformation transitions of those can be visualized by atomic force microscope (AFM) with the capabilities to determine the existence of targets at molecular resolution.

Furthermore, DNA nanorobots can act as an ideal candidate for precision therapeutic applications through a combination of several unique features of tissue penetration, site-targeting, and cargo delivery. Meanwhile, DNA nanorobots have been equipped with various logic gates (AND, OR, XOR, NAND, NOT, CNOT, and a half adder, Figures 1(b) and 1(c)) [10, 11], which can be utilized to display different drug outputs and interface with living systems ranging from cultured cells to mammals. As a powerful toolbox for cancer treatment, DNA nanorobots have been simultaneously loaded with therapeutic cargo and fastened along the periphery by targeting aptamer, so as to explicitly accumulate on tumor sites where in response to molecular trigger the cargo was released to inhibit tumor growth [9]. Because DNA nanorobots are conveniently regulated and biologically amenable, we believe that DNA nanorobots will even enable treating specific tumors via several potential ways like cascade drug delivery, spatiotemporally controlled drug release, and combination with immunotherapy. The last case could for example reprogram immune systems using CpG sequence-rich and/or antigen-modified DNA nanorobots, which will indeed have far-reaching outcomes.

Even though DNA nanorobots have seen immense advancements, their limited stability and behavior in physiologically relevant conditions have raised concerns regarding insufficient circulation time and biodistribution. Consequently, an increasing amount of efforts, such as covalent cross-linking, crossover design, and mineralization, have been invested to increase resistance against disassembly, denaturation, and enzyme digestion [12]. While many of those approaches have indeed resulted in significant improvements in nanorobot's properties in physiological conditions, in most cases, they may be incompatible with a dynamic switching of the DNA nanorobots so as to eventually interfere with the loading and release of therapeutic cargo, binding of diagnostic biomarkers, and diagnostic and/or therapeutic performance. In this context, control over nanorobot's stability, cargo loading and release, analyte binding, and dynamic switching both independently and simultaneously represents the most eminent challenge that biomedical DNA nanorobots currently face.

Typical laboratory synthesis nowadays allows production of nanomole scale of DNA nanorobots for cost of >1000 dollars. Previous successful trail has reported that intravenous administration of DNA nanorobots into a mouse needs about 1 nanomole per dose [13], and the amount for an adult human would translate to be about as large as 300 nanomoles per dose that is prized at as high as >300,000 dollars per dose. Else, such biologics has to comply with CMC and GMP standards due to the issues of sterilization, purification, and batch-to-batch consistency, thus, further increasing the production cost. In this context, scaling up DNA nanorobots with low-cost under CMC and GMP standards represents other pertinent challenges regarding the clinical translation. Although DNA nanorobots still remain miles away from the real-world applications, we can foresee that they would improve human health once those remained challenges are addressed by combining possible scalable and cost-efficient methods of liquid-phase oligonucleotide synthesis [14], chip-based staple strand production [15, 16], DNazyme-catalyzed production [17, 18], and biotechnological “mass production” of DNA nanorobots [19, 20].

## Data Availability

The data used to support the findings of this study are available from the corresponding author upon request.
